# Exploring the relationship between heavy metals and diabetic retinopathy: a machine learning modeling approach

**DOI:** 10.1038/s41598-024-63916-w

**Published:** 2024-06-06

**Authors:** Yanchao Gui, Siyu Gui, Xinchen Wang, Yiran Li, Yueyang Xu, Jinsong Zhang

**Affiliations:** 1 Department of Ophthalmology, Anqing Second People’s Hospital, 79 Guanyuemiao Street, Anqing, 246004 China; 2 Department of Ophthalmology, The Second Affiliated Hospital of Anhui Medical University, 678 Furong Road, Hefei, 230601 China; 3 Department of Clinical Medicine, The Second School of Clinical Medicine, Anhui Medical University, 81 Meishan Road, Hefei, 230032 China; 4 Department of Clinical Medicine, The First School of Clinical Medicine, Anhui Medical University, 81 Meishan Road, Hefei, 230032 China

**Keywords:** Diabetic retinopathy, Heavy metal, Machine learning, Prediction, Factors, Diseases, Medical research, Risk factors

## Abstract

Diabetic retinopathy (DR) is one of the leading causes of adult blindness in the United States. Although studies applying traditional statistical methods have revealed that heavy metals may be essential environmental risk factors for diabetic retinopathy, there is a lack of analyses based on machine learning (ML) methods to adequately explain the complex relationship between heavy metals and DR and the interactions between variables. Based on characteristic variables of participants with and without DR and heavy metal exposure data obtained from the NHANES database (2003–2010), a ML model was developed for effective prediction of DR. The best predictive model for DR was selected from 11 models by receiver operating characteristic curve (ROC) analysis. Further permutation feature importance (PFI) analysis, partial dependence plots (PDP) analysis, and SHapley Additive exPlanations (SHAP) analysis were used to assess the model capability and key influencing factors. A total of 1042 eligible individuals were randomly assigned to two groups for training and testing set of the prediction model. ROC analysis showed that the k-nearest neighbour (KNN) model had the highest prediction performance, achieving close to 100% accuracy in the testing set. Urinary Sb level was identified as the critical heavy metal affecting the predicted risk of DR, with a contribution weight of 1.730632 ± 1.791722, which was much higher than that of other heavy metals and baseline variables. The results of the PDP analysis and the SHAP analysis also indicated that antimony (Sb) had a more significant effect on DR. The interaction between age and Sb was more significant compared to other variables and metal pairs. We found that Sb could serve as a potential predictor of DR and that Sb may influence the development of DR by mediating cellular and systemic senescence. The study revealed that monitoring urinary Sb levels can be useful for early non-invasive screening and intervention in DR development, and also highlighted the important role of constructed ML models in explaining the effects of heavy metal exposure on DR.

## Introduction

Diabetic retinopathy (DR) is the most common microvascular complication of diabetes mellitus and the leading cause of blindness in individuals of working age^1^. The Global Burden of Disease Study, which covered 1990–2020, showed that the global prevalence of diabetic retinopathy has increased by 44. 5% over the past 30 years^2^. Intravitreal injections of anti-vascular endothelial growth factor (anti-VEGF) are the primary disease treatment and management option for patients with advanced DR, but currently there are no diagnostic biomarkers or effective treatments for early-stage DR^3^. Given that the majority of visual impairment and blindness caused by diabetic retinopathy can be avoided by early screening, timely diagnosis of DR and prediction of lesion progression still hold great potential for reducing the incidence of adverse disease outcomes. Although the mechanisms associated with the development of DR are uncertain, current factors such as age, BMI, and gender have been identified as significant risk factors for DR^4^. In addition, researches have revealed that heavy metal exposure is highly correlated with DR risk, suggesting that it may serve as a potentially important predictor of DR^5^.

Heavy metals refer to metals with density greater than 5 g/cm3 and include chromium (Cr), lead (Pb), cadmium (Cd), mercury (Hg), copper (Cu), zinc (Zn), arsenic (As), iron (Fe), cobalt (Co), and manganese (Mn), etc.^6^. Studies have shown that people are permanently exposed to toxic chemicals through air, soil and water pollution, and may also develop chronic bioaccumulation of polyfluoroalkyl substances (PFASs) and multiple heavy metals through the food chain, leading to significant increase in risk of metabolic disease^7,8^. Due to their non-biodegradable toxicity, heavy metals form deposits in the body of humans after prolonged exposure to heavy metals, which cause impairment of brain, liver, kidney, spleen, and eye functions^9^. For example, Shu et al. suggested endoplasmic reticulum stress and impaired function of RPE cells triggered by iron accumulation in retina^10^. Epidemiological studies have also assessed the relationship between heavy metals and diabetes-related adverse events. A Chinese prospective cohort study that followed patients for 3 years and recorded fasting blood glucose and urinary Cd levels reported that each tenfold increase in urinary Cd was associated with a 42% increase in the prevalence of prediabetes^11^. However, a previous study found that serum Cd levels were negatively associated with the risk of diabetes in American people over 20 years old. A NHANES study covering type 2 diabetic patients over 30 years of age in the United States proposed no statistically significant association between blood Cd, selenium (Se), Hg or Pb and DR, and there was even a negative correlation between serum Mn levels and the prevalence of DR in patients with Type 2 Diabetes Mellitus (T2DM)^12^.

Published studies have revealed the association between long-term exposure to heavy metals and elevated risk of DR, but the methodology is still limited to the traditional linear statistical approach, which makes it difficult to adequately explain the complex relationship between heavy metals and DR. Meanwhile the impact of synergistic effects among heavy metals remains a controversial issue^13,14^. Machine learning (ML) techniques are able to incorporate different ranges of data and provide accurate predictions by training and validating models based on the study data^15,16^. ML techniques are widely used in the prediction of disease occurrence and prognosis due to their excellent generalisability and differentiability^17,18^. Several studies have been conducted to predict the risk of diabetes and complications based on ML techniques^16,17,19^. Zhao et al. assessed the risk of diabetic retinopathy in patients with type 2 diabetes mellitus based on five machine learning models, and results showed that the XGBoost model was highly effective in predicting the risk of DR and outperformed the clinical diagnostic methods^20^. However, among the published studies based on machine learning modelling approaches, the prediction of DR risk by environmental factors such as heavy metal exposure is still insufficient, as well as the application of identifying risk factors for DR (e.g., heavy metal exposure, etc.) through interpretable machine learning models.

Traditional statistical methods have some limitations in research, which may affect the reliability and validity of the study. Firstly, traditional statistical methods often rely on linear assumptions about the data, which limits their ability to model complex non-linear relationships. Furthermore, traditional statistical methods often require rigorous testing of premise assumptions on the data before modelling can begin, which may lead to over-simplifying the model or omitting important information from the data. Moreover, traditional statistical methods are often sensitive to outliers and missing values in the data, which can lead to biased or unstable models. In contrast, machine learning methods can provide more flexible and accurate modelling capabilities. Firstly, machine learning methods are able to detect complex non-linear relationships in data automatically, eliminating the need to manually specify the model structure. Second, machine learning methods are able to more flexibly adapt to various types of data including dealing with outliers and missing values in the data without relying on premise assumptions.

The National Health and Nutrition Examination Survey (NHANES) is a cross-sectional survey conducted on various populations in the United States, collecting information on population health, nutrition, and sociodemographic factors. In this study, we utilized heavy metal exposure data from the NHANES surveys conducted between 2003 and 2010 to develop a predictive model for diabetic retinopathy. Additionally, we applied three interpretability methods, including permutation feature importance (PFI) analysis, partial dependence plots (PDP) analysis, and SHapley Additive exPlanations (SHAP) analysis, to the predictive model. The aim is to quantify the impact of different heavy metals and baseline characteristics on the predictive model, investigate the nonlinear relationship between heavy metals and diabetic retinopathy, explore the synergistic effects among them, and formulate effective strategies for controlling diabetic retinopathy based on the predictive model.

## Methods

### Study population

NHANES is a nationwide cross-sectional survey that assesses health and nutritional status through a complex, multistage sampling design. The survey is led by the National Center for Health Statistics, part of the Centers for Disease Control and Prevention (CDC), and is updated every two years, providing data on the U.S. population. Publicly available datasets were analyzed in this study. This data can be found here: https://www.cdc.gov/nchs/nhanes/index.htm. We extracted urine heavy metal concentrations from the four NHANES cycles (2003–2004, 2005–2006, 2007–2008, and 2009–2010), as well as data on the corresponding sociodemographic covariates for the participants, and excluded participants from the analyses if any data were missing.

### Definition of DR and measurement of metal concentrations

DR diagnosis was made by responding to the following question: "Has a doctor ever told you that diabetes has affected your eyes or that you had retinopathy?" Participants were asked to self-report their diabetes diagnosis to include diabetic patients and exclude non-diabetic patients. The diagnosis of DR was further made on the basis of fundus photography results, and diabetic patients were divided into DR group and non-DR group.

Urinary heavy metals (barium (Ba), cadmium (Cd), cobalt (Co), cesium (Cs), molybdenum (Mo), lead (Pb), antimony (Sb), thallium (Tl), tungsten (Tu), uranium (Ur), arsenic (As), mercury (Hg ), and lead (Pb)) were initially assessed as potential predictors of DR. All measurements were performed at the National Center for Environmental Health of the Centers for Disease Control and Prevention (CDC) using the inductively coupled plasma dynamic reaction cell mass spectrometer (ICP-DRC-MS). For more details on laboratory methods and quality control/quality assurance data, please visit the NHANES website.

### Sociodemographic variables

Information about covariates such as age was obtained through interviews or physical examinations (physiological measurements, laboratory tests, etc.). Specifically, race was categorised into four groups: non-Hispanic white, non-Hispanic black, Mexican American, or other. Educational level was categorised as low or high based on whether or not the participant had earned a high school diploma. Economic status was defined as below the poverty line (< 1.00) or above the poverty line (≥ 1.00) based on poverty-to-income ratio (PIR). Marital status was categorised as married, living with a partner or unmarried or other age, in addition to the inclusion of smoking status (never, former, current), alcohol drinking in the past year (mild , moderate, never, former, heavy), body mass index (BMI, kg/m2), total energy intake, and urinary creatinine level.

### Correlation analysis and selection of heavy metals

We used Pearson correlation analyses to assess the correlations between the included heavy metals as well as the baseline variables. We also evaluated the effect of covariance between all covariates in the model using the variance inflation factor (VIF). Multicollinearity was considered to be absent when the VIF value was below 10.

### Construction of the machine learning model

We randomly divide the total dataset into two different subsets, including the training set and a test set, with the former accounting for 79% (N = 833) and the latter accounting for 21% (N = 209), via the fivefold cross-validation sampling method. The training set is then partitioned into five equal-sized subsets, namely folds, which are sequentially used as the test set and the other four folds as the training set, and the model is trained on each training set and validated on the corresponding test set. The performance metrics of each cycle are recorded or averaged, and the performance of each model on different folds is combined to select the optimal model. Prediction on the test set using models that have been evaluated and tuned by cross-validation can provide more comprehensive assessment of the generalisation ability of the models. We used 11 ML techniques including Neural Network (NN), Supported Vector Machine (SVM), Multi-Layer Perceptron (MLP), Gaussian Process (GP), Gradient Boosting Machine (GBM), Logistic Regression (LR), Naive Bayes (NB), XGBoost (XGB), C5.0 Decision Trees (C5.0) and k-nearest neighbour (KNN) , Random Forest (RF) to predict DR risk with training set.

### Evaluation of machine learning model

We further assessed the performance of the 11 models by analysing the following indicators, including the area under the curve (AUC) of the receiver operating characteristic curve(ROC), apparent prevalence, true prevalence, sensitivity, specificity, positive predictive value (PPV), Negative predictive value (NPV), positive likelihood ratio (PLR), negative likelihood ratio (NLR). These evaluation indicators were calculated using R software with Windows operating system based on 1000 test sets via the following R packages: "caret", "randomForest", "pROC", "stats", "epiR", "ggplot2", "dplyr".

### Interpretable methods pipeline of prediction models

After selecting the best performing model based on the evaluation indicators, we used PFI analysis, PDP analysis and SHAP analysis to assess the important covariates and interactions, respectively, that affect the prediction results of the model. PFI analysis and SHAP analysis were used to demonstrate the extent to which each variable contributes to the predictive performance of the model, and to determine which variables have a significant impact on the performance of the model^21^. Interpretable ML methods were executed through the following R packages based on R software: "shapviz", "xgboost", "lime", "caret", "DMwR2", "randomForest", "iml ".

We used PDP analysis to investigate the functional relationships between key metal variables and the predicted risk of DR, as well as interaction analyses to uncover complex relationships between variables that can lead to a better understanding of the predictive performance of the constructed models. PDP analyses can provide intuitive graphical explanations of how the level of the included variables affects changes in predicted outcomes, and also assess the predictive power and stability of the model when based on different variables^22^.

SHAP is generally used by researchers to solve black-box problems associated with ML models and to assess the interaction or synergy of two variables^23^. Specifically, we focused on plotting SHAP dependence plots of the relationship between all variables, including heavy metals, and the predicted risk of DR.

### Statistical analysis

Descriptive statistics for continuous variables were reported as median (Q1,Q3) or mean ± SD, and categorical variables were reported as prevalence (%) and analysed using the Pearson chi-square test for p values. All statistical analyses were performed using R software (R 4.1.2) and statistical significance was defined as a p-value of less than 0.05.

## Results

### Characteristics of the study population

Table 1 demonstrated the general characteristics of the participants with and without diabetic retinopathy in this study. A total of 1,042 American adults were included, of whom 212 were diagnosed with DR and 830 with non-DR. The mean age of the total population was about 62 years. Women comprised 53% of the included people, slightly more than men (47%). Non-Hispanic whites (40%), married (61%), those with high school or above levels of education (60%), never smoking (52%), former alcohol drinking (34%), self-reported history of hypertension (75%) and hyperlipidemia (88%), and those with PIR ≥ 1.00 (78%), respectively, accounted for the largest proportions of the total population. Smoking status, urinary creatinine level (-87.5 vs 103) and mean concentrations of heavy metal, such as Sb (-7.30 vs -7.42), Tl (-6.71 vs -6.56) and Pt (-9.41 vs -9.58) differed significantly between DR and non-DR patients.

**Table 1:** Baseline characteristics of Participants.

Characteristic	Study Participants, N (%)/ Mean ± SD/ Median (Q1,Q3)			
	All (n = 1042)	No (n = 830)	Yes (n = 212)	*P*
**Year, y**				0.262
2003–2004	94(9)	74(9)	20(9)	
2005–2006	238(23)	182(22)	56(26)	
2007–2008	338(32)	266(32)	72(34)	
2009–2010	372(36)	308(37)	64(30)	
**Ba, ng/mL**	− 4.61(− 5.21, − 3.92)	− 4.57(− 5.17, − 3.94)	− 4.74(− 5.57, − 3.84)	0.058
**Cd, ng/mL**	− 5.7 ± 0.77	− 5.68 ± 0.76	− 5.79 ± 0.81	0.062
**Co, ng/mL**	− 5.77(− 6.16, − 5.38)	− 5.78(− 6.15, − 5.39)	− 5.72(− 6.16, − 5.28)	0.255
**Cs, ng/mL**	− 3.09(− 3.45, − 2.75)	− 3.1(− 3.43, − 2.75)	− 3.06(− 3.5, − 2.72)	0.976
**Mo, ng/mL**	− 0.76(− 1.17, − 0.33)	− 0.78(− 1.21, − 0.33)	− 0.67(− 1.09, − 0.36)	0.221
**Pb, ng/mL**	− 5.11(− 5.51, − 4.67)	− 5.1(− 5.5, − 4.65)	− 5.16(− 5.56, − 4.67)	0.427
**Sb, ng/mL**	− 7.41(− 7.72, − 6.93)	− 7.42(− 7.72, − 6.97)	− 7.3(− 7.76, − 6.68)	0.012
**Tl, ng/mL**	− 6.59(− 6.95, − 6.2)	− 6.56(− 6.92, − 6.18)	− 6.71(− 7.1, − 6.31)	0.001
**Tu, ng/mL**	− 7.1(− 7.58, − 6.57)	− 7.11(− 7.59, − 6.55)	− 7.01(− 7.55, − 6.65)	0.503
**Ur, ng/mL**	− 9.54(− 10, − 8.92)	− 9.53(− 10, − 8.93)	− 9.56(− 10, − 8.64)	0.657
**Pt, ng/mL**	− 9.53(− 10.04, − 8.83)	− 9.58(− 10.07, − 8.83)	− 9.41(− 9.81, − 8.84)	0.029
**Hg, ng/mL**	− 5.52(− 6.11, − 4.9)	− 5.52(− 6.1, − 4.87)	− 5.53(− 6.14, − 4.94)	0.968
**As, ng/mL**	− 2.42(− 2.96, − 1.74)	− 2.45(− 2.95, − 1.81)	− 2.36(− 3, − 1.56)	0.224
**UCr, (mg/min)**	101(62, 151)	103(62, 154)	87.5(60.75, 135)	0.008
**Age, y**	62(52, 71)	62(52, 72)	61(50, 69.25)	0.23
**Sex**				0.949
Male	487(47)	387(47)	100(47)	
Female	555(53)	443(53)	112(53)	
**Ethnics**				0.131
Mexican American	228(22)	184(22)	44(21)	
Non-Hispanic Black	263(25)	206(25)	57(27)	
Non-Hispanic White	418(40)	343(41)	75(35)	
Other	133(13)	97(12)	36(17)	
**Marital**				0.936
Married or living with a partner	639(61)	510(61)	129(61)	
Unmarried or other	403(39)	320(39)	83(39)	
**Education level**				0.172
Less than High school	412(40)	319(38)	93(44)	
High School or above	630(60)	511(62)	119(56)	
**BMI, kg/m**^**2**^	31.27(27.83, 36.39)	31.26(27.8, 36.26)	31.29(27.87, 37.79)	0.416
**Smoke status**				0.014
Current	159(15)	140(17)	19(9)	
Never	541(52)	419(50)	122(58)	
Former	342(33)	271(33)	71(33)	
**Past-year alcohol drinking**				0.095
Mild	283(27)	221(27)	62(29)	
Moderate	81(8)	62(7)	19(9)	
Never	202(19)	157(19)	45(21)	
Former	357(34)	284(34)	73(34)	
Heavy	119(11)	106(13)	13(6)	
**Hypertension**				0.594
No	263(25)	213(26)	50(24)	
Yes	779(75)	617(74)	162(76)	
**Hyperlipidemia**				1
No	130(12)	104(13)	26(12)	
Yes	912(88)	726(87)	186(88)	
**HEI.score**	50.64(40.81, 61.12)	51.08(40.86, 61.29)	49.34(40.66, 61.11)	0.414
**Poverty ratio**				0.103
At or above poverty line (≥ 1.00)	225(22)	170(20)	55(26)	
Below poverty line (< 1.00)	817(78)	660(80)	157(74)	
**Kcal, kcal/d**				0.153
Very Low (< 1200)	203(19)	173(21)	30(14)	
Low (1200–1600)	205(20)	155(19)	50(24)	
Medium (1600–2000)	215(21)	169(20)	46(22)	
High (2000–2400)	199(19)	155(19)	44(21)	
Very High (> 2400)	220(21)	178(21)	42(20)	

All proportions, means, and SEs are weighted estimates of the US population characteristics, taking into account the complex sampling design of the National Health and Nutrition Examination Survey.

*UCr* urinary creatinine, *BMI* body mass index (calculated as weight in kilograms divided by height in meters squared).

### Correlation analysis and variable selection

**Figure 1:**
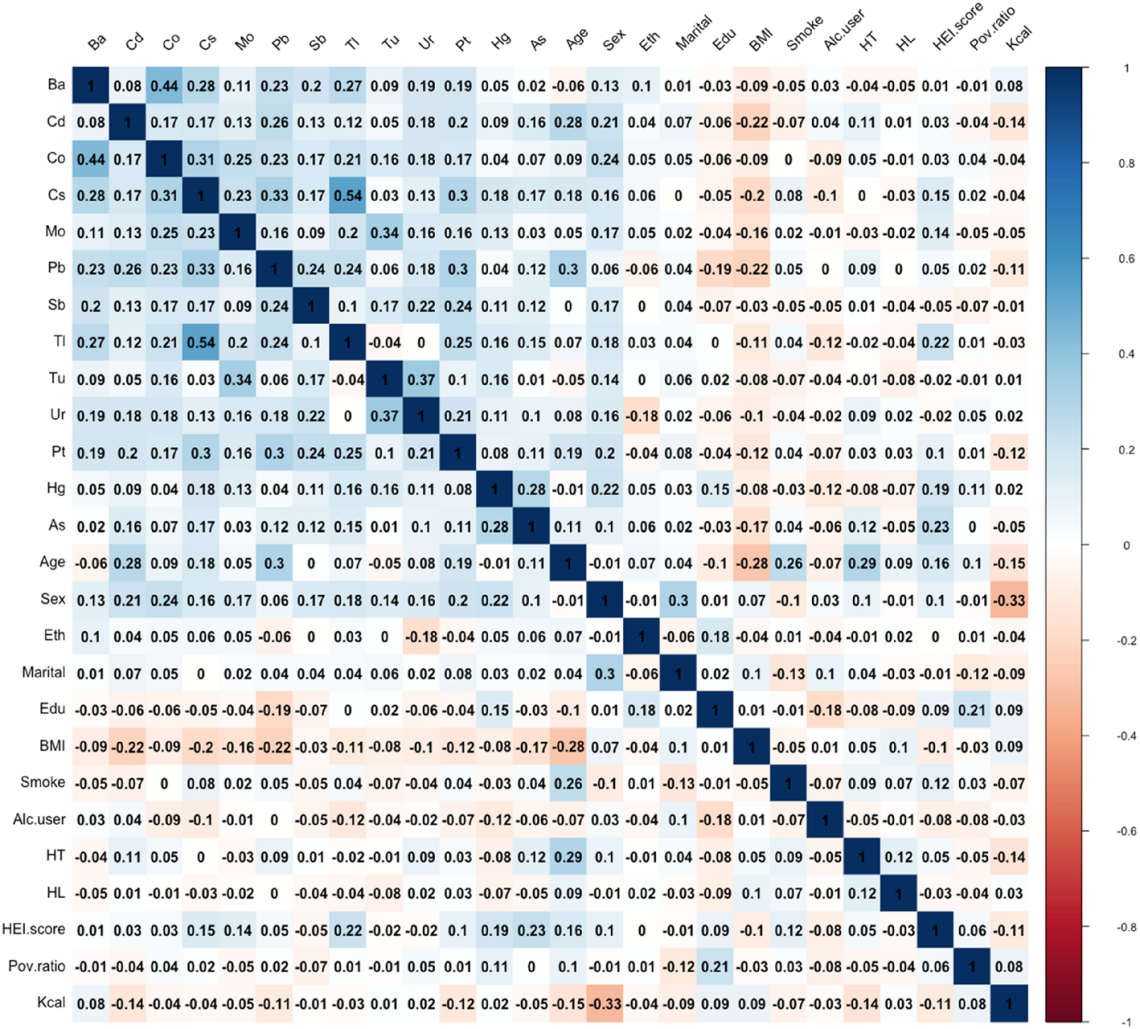
The results of Pearson's correlation analysis among the metal factors and baseline variables.

Figure 1 showed the correlation between each of the 13 heavy metals and the baseline characteristics of the population. The results indicated that most of the metals are correlated with each other to varying degrees, with a relatively strong correlation between TI and Cs (r = 0.54) and the similar relationship between Co and Ba (r = 0.44). Curves based on correlations of heavy metals with baseline population characteristics and other details are shown in Fig. S1. We also assessed multicollinearity between all selected metals and covariates using variance inflation factors (VIFs), which showed that there was no multicollinearity.

### Evaluation and comparison of 11 machine learning models

**Figure 2:**
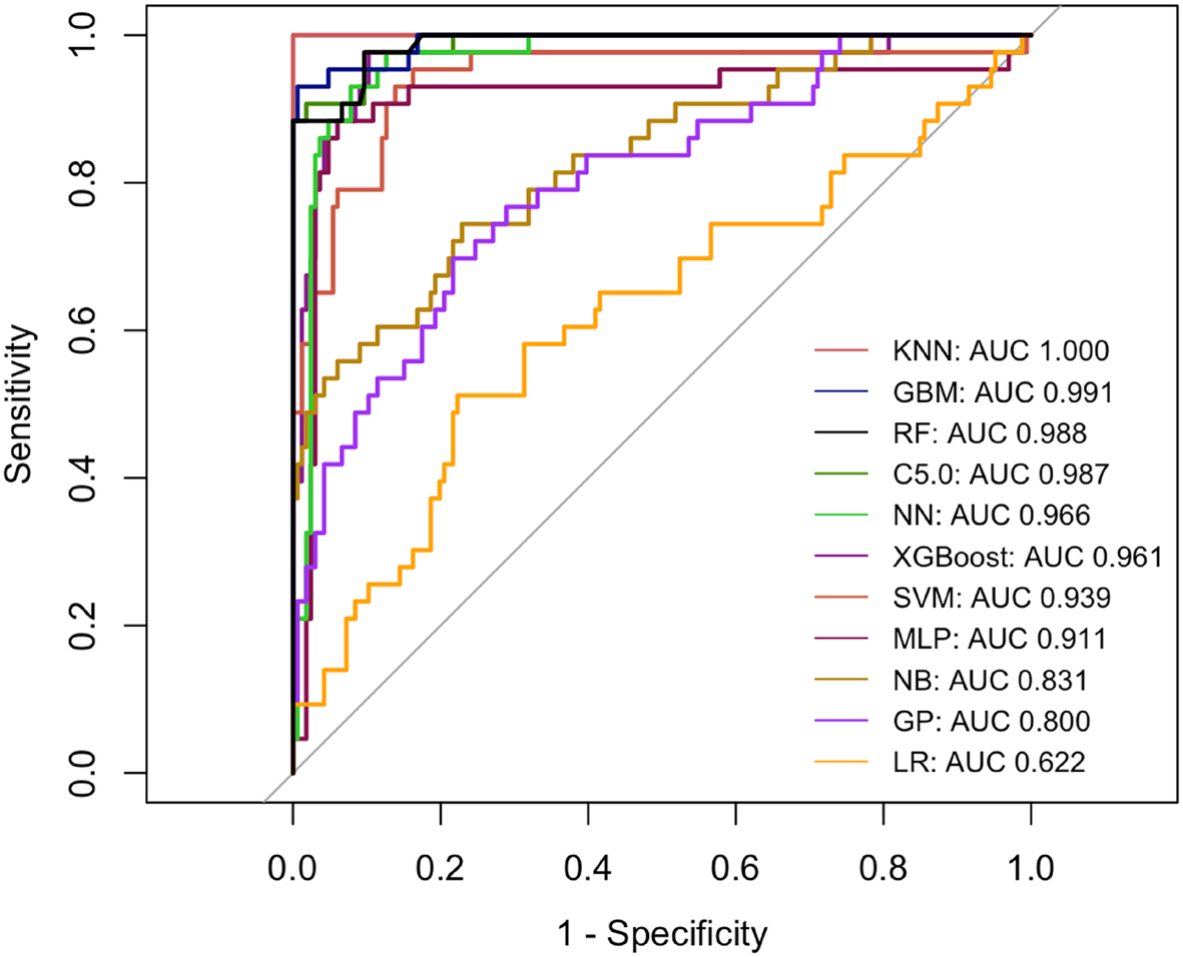
The ROC of the 11 machine learning models in testing set.

Figure 2 showed the efficacy of the 11 machine learning models included to predict DR risk based on the testing set, and the results of the training set are shown in Fig. S2, presented as ROC analysis curves. The AUC value of the KNN model is 1.000, of the GBM model is 0.991, of the RF model is 0.988, of the C5.0 model is 0.987, of the NN model is 0.966, of the XGBoost model is 0.961, of the SVM model is 0.939, of the MLP model is 0.911, of the NB model is 0.831, of the GP model is 0.800, of the LR model is 0.622. Tables S1 and Tables 2 provide the perfomance indicators of the 11 models used in this study in the training set and validation set, respectively, and show the confusion matrices used by 11 machine learning algorithms. The results show that among these machine learning models, the KNN model exhibits the best prediction performance. As a result, the prediction model based on the KNN model was finally selected for subsequent analyses.

**Table 2:** Comparison of discriminative features of 11 machine learning models in testing set.

Characteristics	SVM	NN	MLP	GP	GBM	LR	NB	XGB	C5.0	KNN	RF
Apparent prevalence	0.21(0.08, 0.41)	0.21(0.08, 0.41)	0.25(0.11, 0.45)	0.14(0.06, 0.27)	0.78(0.71, 0.83)	0.14(0.06, 0.27)	0.43(0.24, 0.63)	0.18(0.09, 0.31)	0.18(0.06, 0.37)	0.21(0.08, 0.41)	0.82(0.76, 0.87)
True prevalence	0.32(0.16, 0.52)	0.32(0.16, 0.52)	0.32(0.16, 0.52)	0.18(0.09, 0.31)	0.79(0.73, 0.85)	0.18(0.09, 0.31)	0.32(0.16, 0.52)	0.18(0.09, 0.31)	0.32(0.16, 0.52)	0.32(0.16, 0.52)	0.79(0.73, 0.85)
Sensitivity	0.56(0.21, 0.86)	0.56(0.21, 0.86)	0.67(0.30, 0.93)	0.56(0.21, 0.86)	0.96(0.92, 0.98)	0.56(0.21, 0.86)	0.89(0.52, 1.00)	0.78(0.40, 0.97)	0.56(0.21, 0.86)	0.56(0.21, 0.86)	1.00(0.98, 1.00)
Specificity	0.95(0.74, 1.00)	0.95(0.74, 1.00)	0.95(0.74, 1.00)	0.95(0.83, 0.99)	0.93(0.81, 0.99)	0.95(0.83, 0.99)	0.79(0.54, 0.94)	0.95(0.83, 0.99)	1.00(0.82, 1.00)	0.95(0.74, 1.00)	0.88(0.75, 0.96)
PPV	0.83(0.36, 1.00)	0.83(0.36, 1.00)	0.86(0.42, 1.00)	0.71(0.29, 0.96)	0.98(0.95, 1.00)	0.71(0.29, 0.96)	0.67(0.35, 0.90)	0.78(0.40, 0.97)	1.00(0.48, 1.00)	0.83(0.36, 1.00)	0.97(0.93, 0.99)
NPV	0.82(0.60, 0.95)	0.82(0.60, 0.95)	0.86(0.64, 0.97)	0.91(0.78, 0.97)	0.85(0.72, 0.94)	0.91(0.78, 0.97)	0.94(0.70, 1.00)	0.95(0.83, 0.99)	0.83(0.61, 0.95)	0.82(0.60, 0.95)	1.00(0.91, 1.00)
PLR	10.56(1.44, 77.62)	10.56(1.44, 77.62)	12.67(1.78, 90.18)	11.39(2.61, 49.66)	13.73(4.61, 40.91)	11.39(2.61, 49.66)	4.22(1.72, 10.39)	15.94(3.95, 64.40)	Inf(NaN, Inf)	10.56(1.44, 77.62)	8.60(3.77, 19.60)
NLR	0.47(0.22, 0.98)	0.47(0.22, 0.98)	0.35(0.14, 0.89)	0.47(0.22, 0.97)	0.05(0.02, 0.09)	0.47(0.22, 0.97)	0.14(0.02, 0.91)	0.23(0.07, 0.79)	0.44(0.21, 0.92)	0.47(0.22, 0.98)	0.00(0.00, NaN)

All constructed predictive models were developed without the utilization of data augmentation techniques.

*SVM* supported vector machine, *NN* neural network, *MLP* multi-layer perceptron, *GP* gaussian process, *GBM* gradient boosting machine, *LR* logistic regression, *NB* Naive Bayes, *XGB* XGBoost, *C5.0* C5.0 Decision Trees, *KNN* k-nearest neighbor, *RF* random forest, *PPV* positive predictive value, *NPV* negative predictive value, *PLR* positive likelihood ratio, *NLR* negative likelihood ratio.

### Interpretable methods pipeline

#### Key metals among the important variables

**Figure 3:**
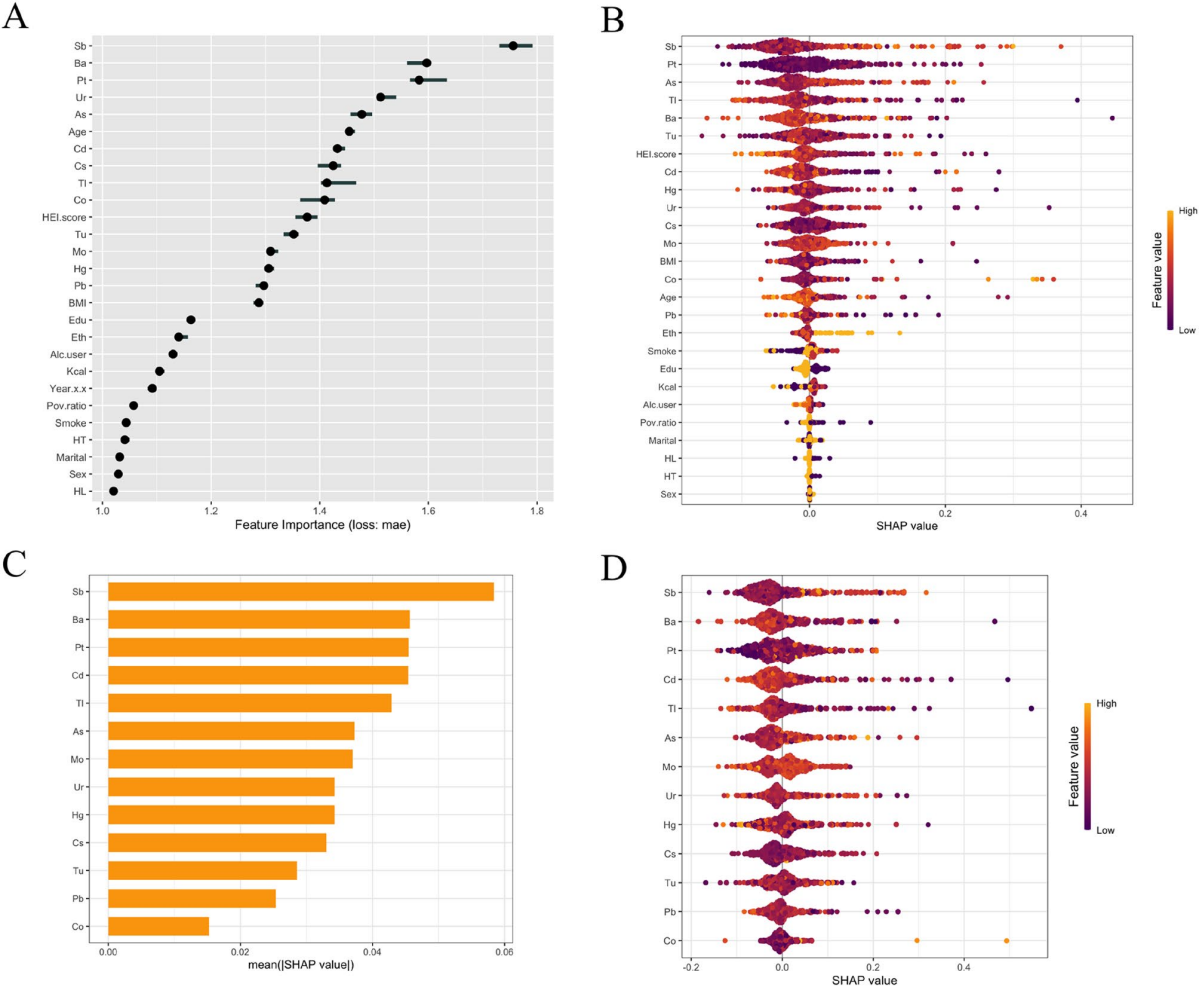
The contribution of metal factors and baseline variables in predictive model. (**A**) The forest map based on PFI analysis displays the corresponding contribution weights of heavy metals and baseline variables and their corresponding standard deviations; (**B**) The SHAP summary plot of all variables and DR risk. The width of the range of the horizontal bars can be interpreted as the effect on the model predictions, with the wider the range, the greater the effect. The direction on the x-axis represented the likelihood of developing DR (right) or not developing (left); (**C**) The SHAP features importance plot of heavy metals and DR risk. The magnitude of the effect of each feature on the model output was measured by the average of the absolute values of the SHAP values for all samples, ranked from top to bottom by their magnitude of effect; D) The SHAP summary plot of heavy metals and DR risk.

PFI analysis provided insights into the relative importance of all variables in the KNN model. We used the IML method to assess the contribution weights of heavy metal exposure (Ba, Cd, Co, Cs, Pb, Sb, etc.) and people's baseline characteristics (age, sex, BMI, education level, ethnicity, smoking and drinking status, etc.) in the prediction model, which is presented in Fig. 3A. The results of the analyses showed that the first five variables (Sb, Ba , Pt, Ur, As) were relatively more important variables in the prediction model. Among them, Sb level contributed a weight of 1.730632 ± 1.791722 in predicting DR risk, which was significantly higher than all other included variables. The critical variables only compared to Sb level also include Ba, Pt, Ur, As, which are also relatively sensitive metals in predicting the development of DR. The contribution weights of Ba, Pt, Ur, As were 1.560474 ± 1.602271, 1.566063 ± 1.633790, 1.511366 ± 1.540538, 1.456352 ± 1.496473 respectively. It is worth noting that the contribution weights of demographic characteristics and lifestyle-related variables in the prediction of DR risk in our results were lower compared to heavy metal exposure, and all baseline characteristics variables except age were weaker than heavy metal exposure.

Furthermore, we further validated the relationship of each variable with the predicted DR risk by the SHAP method after screening the KNN model. The SHAP summary plot (Fig. 3B) showed the overall effect of heavy metals and baseline variables on DR risk, and was ranked in descending order according to the importance of the feature. In this case, a positive SHAP value indicates that the value of the feature is positively associated with DR risk, and the larger the value, the greater the contribution. The results showed that the top five potentially critical factors influencing higher predicted DR risk were, in descending order, Sb, Pt, As, Tl, Ba. Moreover, Sb had higher contribution weight in the prediction model than any other heavy metal or baseline variable under two different analysis methods, which is in line with the results of the SHAP summary plot between heavy metals and predicted DR risk (Fig. 3C,D).

#### Relationships between the key metals and DR

**Figure 4:**
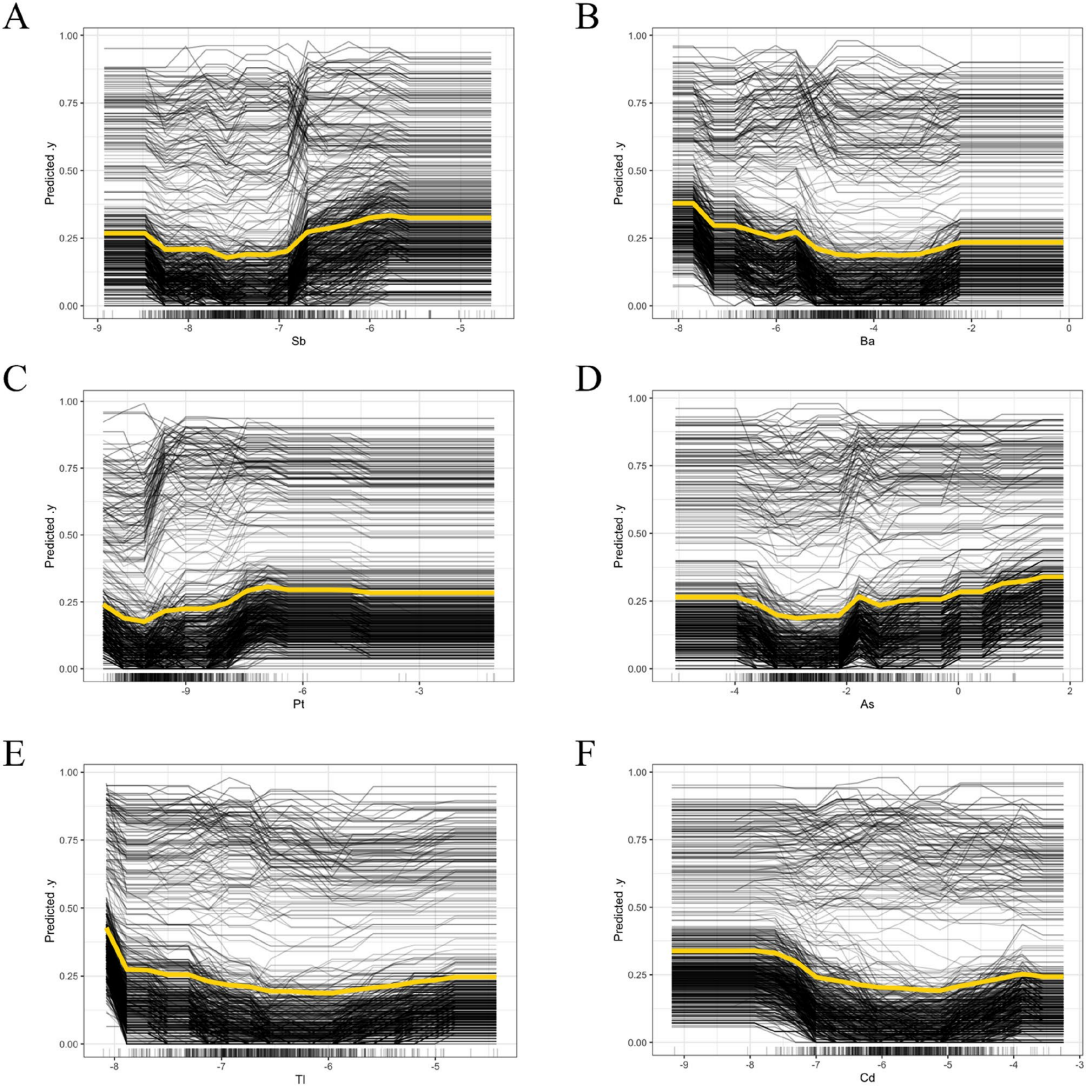
Relationships between key metal including (**A**) Sb, (**B**) Ba, (**C**) Pt, (**D**) As, (**E**) Tl, (**F**) Cd and predictive DR risk. The x-axis of the plot represented the log-transformed values of each metal.

The predictive performance of the selected KNN model was further explained by PDP analysis, and the relationships between six key heavy metals (Sb, Ba, Pt, As, Tl, Cd) and the predicted values of DR are shown in Fig. 4, while the results for the remaining heavy metals are shown in Fig. S3. The results show that some of the heavy metals, including As, Co, Sb, and Tu, showed a significant trend of increasing predicted risk of DR with elevated levels of these heavy metals in the log-transformed interval of the relatively high concentrations. The predicted risk of DR was significantly increased when the log-transformed levels of some heavy metals, including As, Co, Sb, and Tu, were elevated at relatively high concentration, but there was no significant correlation between Pt and the predicted risk of DR at high concentration. However, there was no significant correlation between increasing or decreasing levels of Cs, Hg, and Pb and DR risk. These findings suggest that timely detection of key metal levels in vivo may play an essential role in predicting the development of DR.

#### Interaction effects of variables on DR

**Figure 5:**
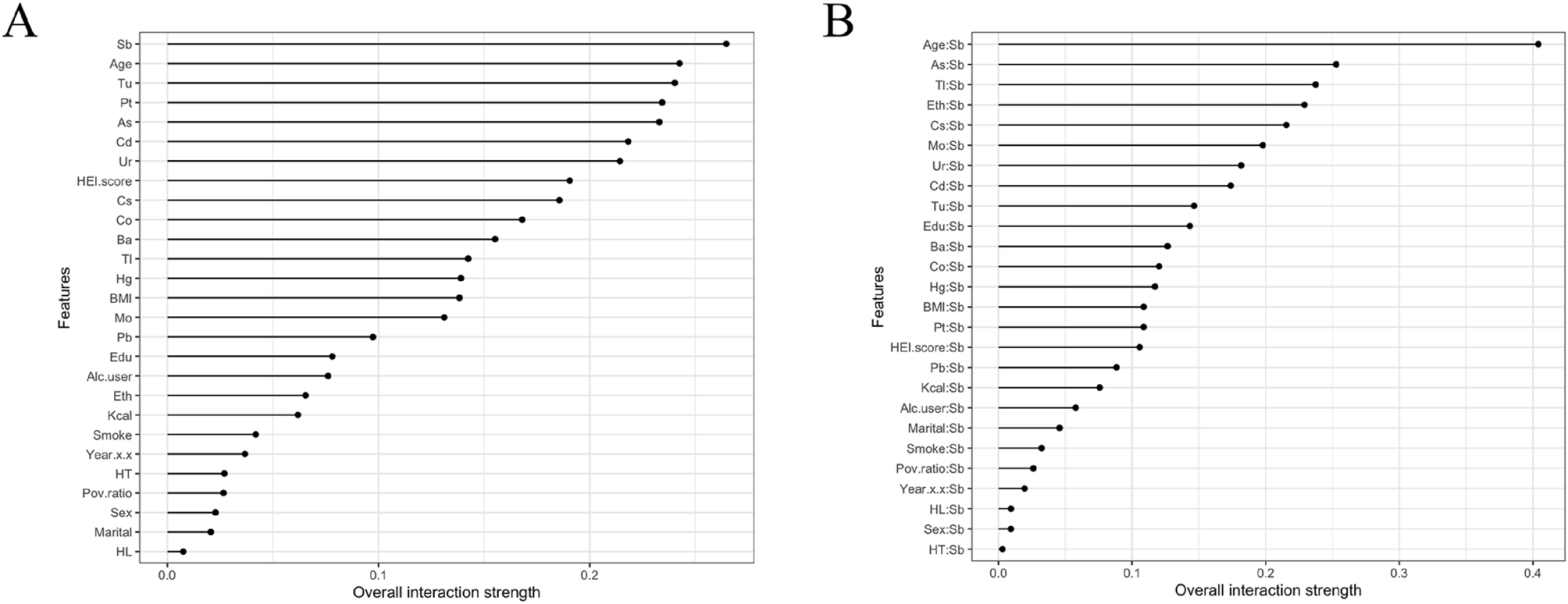
Interaction effects of variables on DR. (**A**) Interactions between heavy metals and baseline variables on DR; (**B**) Interactions between Sb levels and other variables on DR. The range of the straight line represents the overall interaction strength, the wider the range, the greater the effect.

We performed the analysis of heavy metal exposure interaction properties by PDPs model. The results in Fig. 5A show that the corresponding variables with overall interaction strength greater than 0.2 were Sb, age, Tu, Pt, As, Cd, and Ur, with Sb having the most significant interaction effect. The interaction performance of the baseline variables for the prediction of DR risk remained weaker than that of heavy metals. Therefore, we further performed the interaction analysis of Sb with other variables. Figure 5B revealed that the interaction between Sb and age ranked the highest among all metal pairs, with overall interaction strength greater than 0.4. In addition to the strong synergistic effect of Sb with As, Tl, and Cs, ethnicity had an effect on the prediction of DR risk by Sb, with overall interaction strength greater than 0.2. The results suggest that monitoring Sb levels, especially in older populations, may be more critical in controlling the development of DR.

## Discussion

Using heavy metal exposure data from the National Health and Nutrition Examination Survey (NHANES) between 2003 and 2010, we used 11 machine learning modeling techniques to develop a model that could more accurately predict the risk of DR, along with better understanding of the relationship and interactions between heavy metal exposure and DR. Specifically, we selected the best-performing KNN model for the follow-up study through survival analysis, and further assessed the contribution weights of heavy metal exposure and demographic characteristics in predicting DR risk by PFI analysis and SHAP analysis, and we found that urinary Sb levels had the most significant effect on the prediction of DR risk compared to other variables . PDP analysis fitted the non-linear relationship between the variables included in the study and the predicted value of DR. Moreover, we performed interaction analysis of heavy metal exposure with baseline variables.

The development of machine learning techniques has somewhat remedied some of the methodological shortcomings of previous prediction models of environmental exposures associated with various adverse health outcomes^4^. In recent years, with the gradual and widespread application of machine learning models, heavy metals, as important environmental exposure risk factors for human health, have shown good predictive potential in predicting disease outcomes and complications, such as renal damage^24^, osteoarthritis^25^ and type 2 diabetes^16^. To the best of our knowledge, only two studies have used machine learning techniques for the construction of DR risk prediction models. Zhao et al.^20^ assessed the risk of diabetic retinopathy in patients with type 2 diabetes based on five machine learning models, and showed that the XGBoost model predicted the risk of DR significantly and exceeded the clinical diagnostic methods for DR. Zong et al. also succeeded in building well-performing predictive DR risk models through machine learning techniques {Zong, 2023 #14}. Both studies explored non-environmental risk factors that may be associated with diabetic retinopathy, but both ignored the effects of environmental exposures.

Methods applied in this study such as PFI analysis, based on a reliable and well-performing predictive model to rank permutation feature importance of included variables in influencing disease prediction, in order to identify potential critical predictors^26,27^. PDP analysis revealed the relationship between individual variables and predicted DR risk as well as interactions among variables based on the KNN model^27^. Moreover, Feng et al. applied the same methodology in the study and successfully demonstrated potential functional association between chronic cadmium accumulation and severity of renal damage^24^. In a predictive model developed using machine learning techniques, we found association between heavy metal exposure and DR risk and revealed that Sb is the critical metal element by PFI analysis, PDP analysis and SHAP analysis.

Most toxic metals are widely distributed in industrial products, soil, food, and drinking water, and accumulate in the human liver, kidneys, muscles, and eyes due to their non-degradability^28^. Patients with DR have been detected with higher concentrations of heavy metals such as Cd and Co in the eye compared to healthy individuals of the same age^29^. Sb, which is mainly used in the manufacture of alloys and semiconductor materials, is a global pollutant and one of the toxic metallic elements of greatest global concern. Sb is toxic to the human body mainly by binding to sulfhydryl groups in human cells and inactivating related proteins and enzymes in the body. We found significant positive correlation between high levels of Sb and predicted risk of DR by PDP analysis. The Los Angeles Cohort Study of Low-Income Hispanic Pregnancies suggests that urinary Sb levels in pregnant women during early pregnancy are the highest in comparison to Hg and Ni in the ranking of predictors of gestational age for birth weight^30^. The New Hampshire birth cohort study also found that prenatal exposure of pregnant women to certain toxic metals, such as Sb, may have deleterious effects on fetal lung function^31^. Compared to other toxic metals such as mercury and arsenic, there is a lack of systematic understanding of the environmental contamination process of Sb. To the best of our knowledge, there are no relevant studies focusing on Sb and the development of DR, and the evidence from population-based studies on this topic, especially in ocular diseases, is very limited. However, the results of this study show the importance of Sb levels in the predictive performance of the machine learning model we constructed, combined with the results of epidemiological studies related to other diseases as well as the toxicity of Sb itself, suggest that the public should be more concerned about this heavy metal with chronic toxicity.

The hyperglycaemic environment of the eyes in diabetic patients leads to an overproduction of reactive oxygen species (ROS), triggering the inflammatory response and altered vascular permeability. The cytotoxicity of Sb disrupts the intracellular redox balance, causing abnormally high levels of ROS and cellular dysfunction^32,33^. Furthermore, it has been found that both early and late AMD patients have significantly higher levels of As in the retina than healthy individuals of the same age group^34^. Zhong et al. showed that deposition of As and Sb after mixed exposure induces intracellular mitochondrial damage and promotes cell apoptosis^33^. On the other hand, As, Cd and Pb mixtures have been shown to promote rat retinal ganglion cell apoptosis and result in decrease in brain-derived neurotrophic factor, which induces abnormal development of myelin sheaths and axons in the optic nerve and retina^35^. These findings suggest that exposure to Sb and As is associated with increased risk of retinopathy, consistent with our findings. However, the pathophysiological mechanisms by which Sb and As promote the development of DR are unknown and research evidence is limited.

We found that age has an effect on predicting the risk of diabetic retinopathy by the KNN model, especially the significant interaction with the Sb level, which has the largest contributing weight. With the biological aging of human functions, the immune and defence mechanisms of the retina undergo degenerative changes, leading to increased risk of various age-related diseases^36^. A prospective study showed that people aged 50 to 59 years had a higher prevalence of diabetic retinopathy compared with younger people^37^. Aging promotes neutrophil recruitment and pro-inflammatory cytokine overexpression in the retina, exacerbating the inflammatory response, leading to impaired retinal barrier function and increased incidence of neovascularisation, thereby raising DR risk^38^. Furthermore, cellular senescence can induce mitochondrial ROS leakage and endoplasmic reticulum stress through aberrant metabolism, resulting in apoptosis and further dysfunction of cells, which progressively evolve into irreversible lesions in diabetic retinopathy^39^. A NHANES study evaluating urinary heavy metal levels and risk of osteoarthritis found that Cd, Co and Cs accelerate cellular senescence by exacerbating telomere length wear and tear^40^.

Our study emphasized the integration of ML methods with PFI analysis, PDP analysis and SHAP analysis to investigate the impact of environmental exposures on health outcomes. We found that Sb levels were the most significant critical factor influencing the prediction of DR outcomes compared to other variables, and the interaction effect of Sb with age was significantly higher than that of other heavy metals or baseline variables, suggesting that Sb may influence the development of DR by mediating cellular and systemic senescence. In addition, the DR risk prediction model developed in this study may provide effective clinical management and control options for early noninvasive screening, diagnosis, intervention, to effectively avoid visual impairment and blindness caused by advanced DR lesions.

However, there are limitations to this study. Firstly, the inclusion of relevant independent variables, especially demographic baseline variables, in the ML models was not fully considered. In addition, although the performance of the KNN model was satisfactory, the variety of machine learning models we included was still limited and the validation analysis of the model performance was not comprehensive. Finally, despite the good predictive results of the present model, further studies, such as prospective studies to validate in other populations and mechanical studies investigating the intrinsic link between heavy metal exposure and diabetic retinopathy, are needed to validate the clinical applicability.

## Conclusion

Data related to heavy metal exposure and DR diagnosis in NHANES were included to examine the predictive performance and applicability of 11 ML models separately, and the KNN model that could predict DR risk more accurately was chosen to further analysis. We found the critical heavy metals for predicting the risk of DR by permutation feature importance analysis, PDP analysis and SHAP analysis, and observed the relationship between the range of exposure levels of each included heavy metal and the predicted value of DR, as well as the interaction of each variable with DR.

### Supplementary Information

Supplementary Information.

## Data Availability

Publicly available datasets were analyzed in this study. This data can be found here: https://www.cdc.gov/nchs/nhanes/index.htm.
