# Supplementary material for: Exploring the relationship between heavy metals and diabetic retinopathy: a machine learning modeling approach
Source: Sci Rep. 2024 Jun 6;14:13049. doi: 10.1038/s41598-024-63916-w (PMC11156935; doi:10.1038/s41598-024-63916-w)
Supplement: Supplementary file 1 — Supplementary Information. [file 41598_2024_63916_MOESM1_ESM.docx]

**Supplementary materials**

**Exploring the Relationship between Heavy Metals and Diabetic Retinopathy: A Machine Learning Modeling Approach**

**Figure S1.** The results of Pearson's correlation analysis among the metal factors and baseline variables.

**Figure S2.** The ROC of the 11 machine learning models in training set.

**Figure S3.** Relationships between key metal including (A) Tu, (B) Pb, (C) Mo, (D) Co, (E) Hg, (F) Cs and predictive DR risk.

**Table S1.** Comparison of discriminative features of 11 ML models in trainng set.


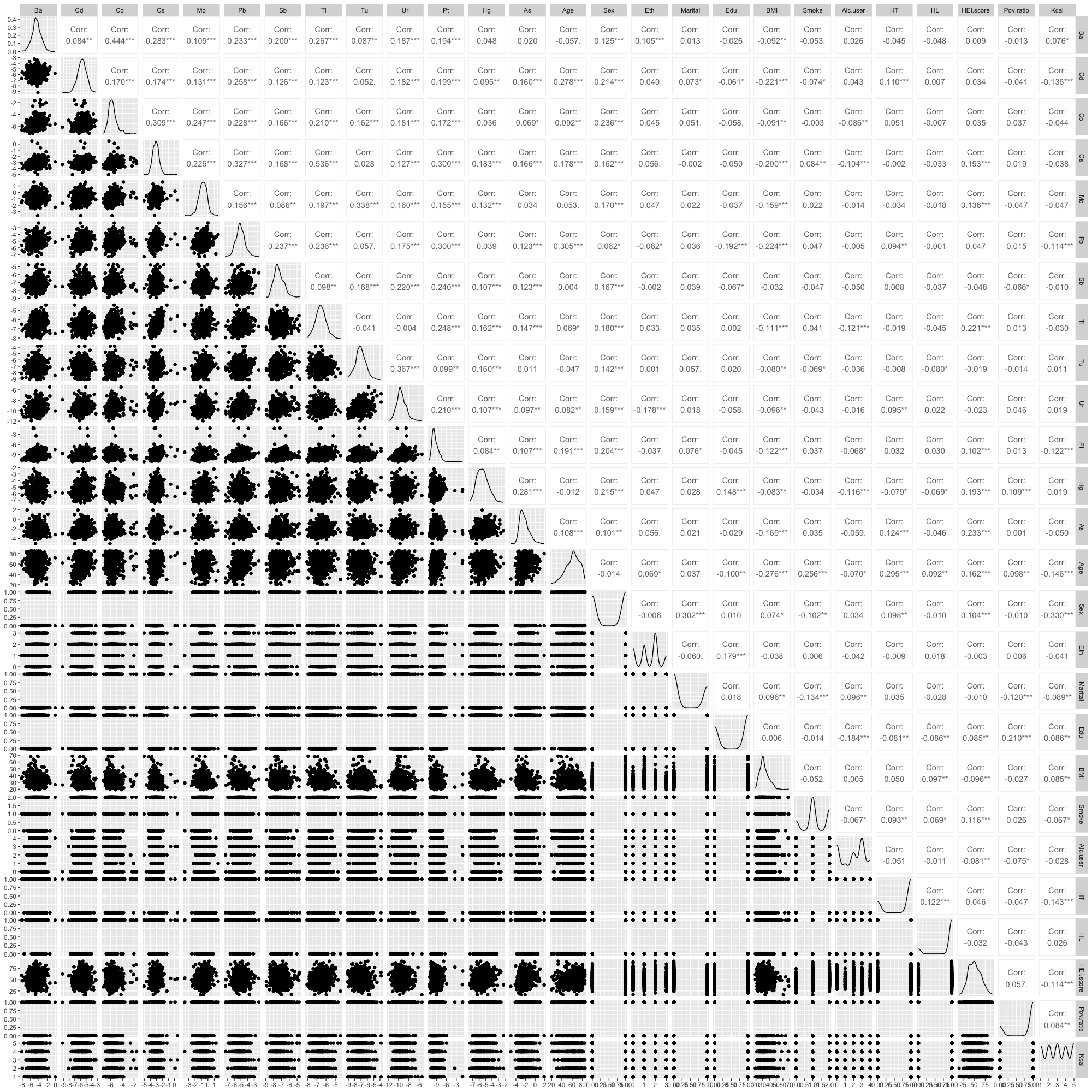


**Figure S1.** The results of Pearson's correlation analysis among the metal factors and baseline variables.

**
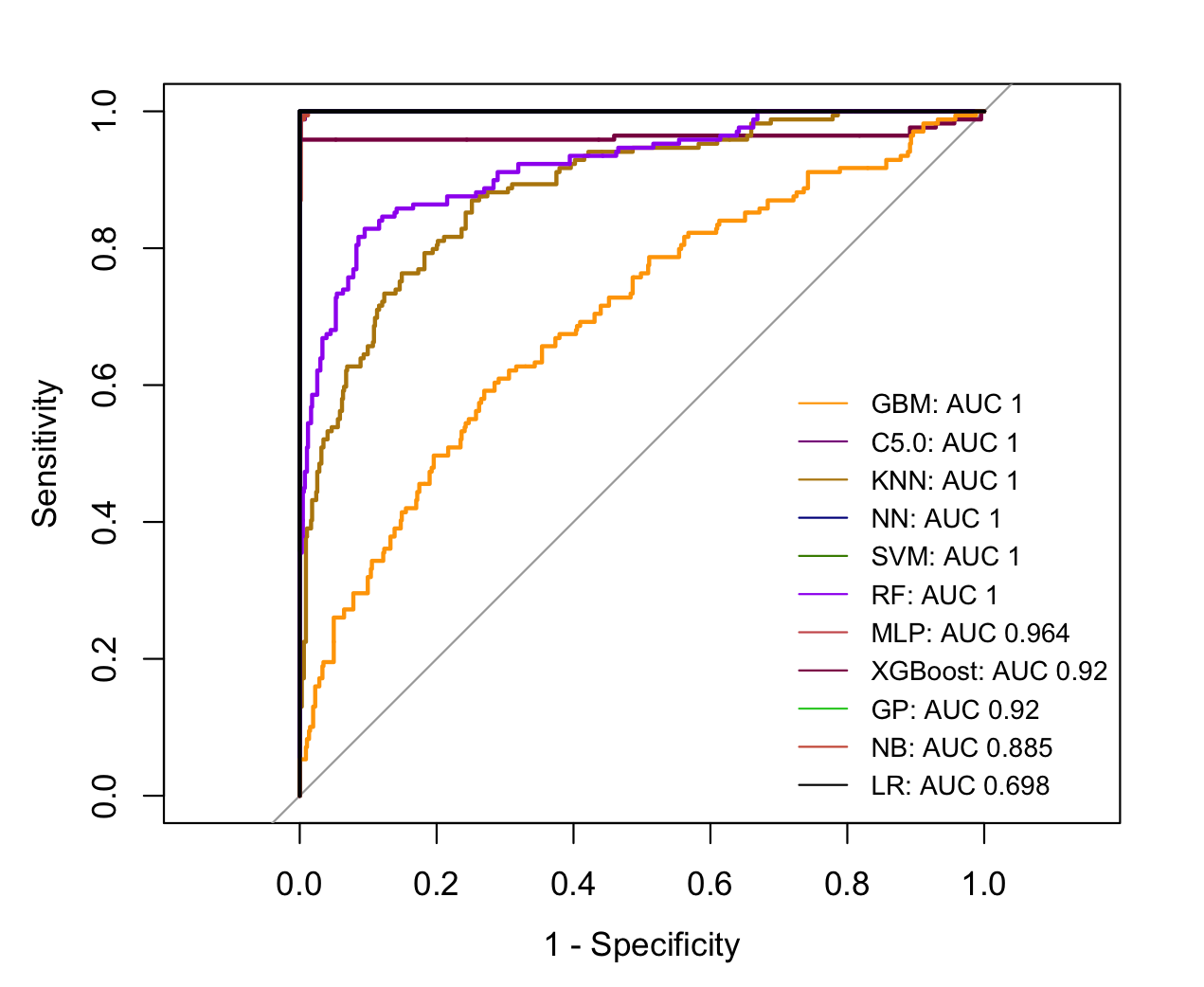
**

**Figure S2.** The ROC of the 11 machine learning models in training set.

**
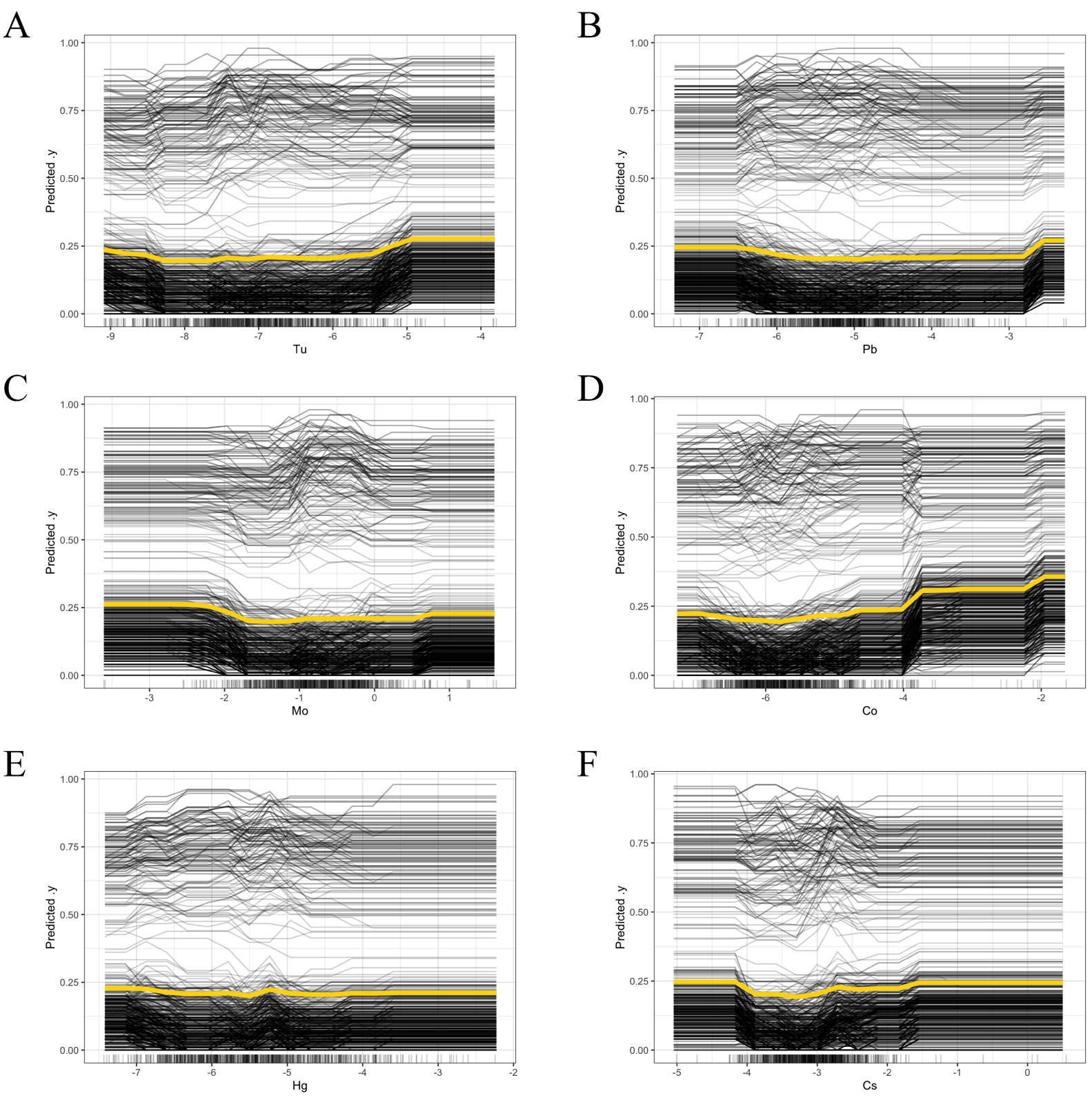
**

**Figure S3.** Relationships between key metal including (A) Tu, (B) Pb, (C) Mo, (D) Co, (E) Hg, (F) Cs and predictive DR risk. The x-axis of the plot represented the log-transformed values of each metal.

**Table S1.** Comparison of discriminative features of 11 ML models in training set ^a^.

| **Characteristics** | **SVM** | **NN** | **MLP** | **GP** | **GBM** | **LR** | **NB** | **XGB** | **C5.0** | **KNN** | **RF** |
| --- | --- | --- | --- | --- | --- | --- | --- | --- | --- | --- | --- |
| **Apparent prevalence** | 0.44(0.34, 0.53) | 0.25(0.18, 0.34) | 0.34(0.25, 0.43) | 0.35(0.26, 0.44) | 0.80(0.77, 0.82) | 0.39(0.30, 0.49) | 0.64(0.55, 0.73) | 0.39(0.30, 0.49) | 0.31(0.23, 0.41) | 0.35(0.26, 0.44) | 0.80(0.77, 0.82) |
| **True prevalence** | 0.38(0.29, 0.48) | 0.49(0.40, 0.57) | 0.38(0.29, 0.48) | 0.38(0.29, 0.48) | 0.80(0.77, 0.82) | 0.38(0.29, 0.48) | 0.38(0.29, 0.48) | 0.38(0.29, 0.48) | 0.38(0.29, 0.48) | 0.38(0.29, 0.48) | 0.80(0.77, 0.82) |
| **Sensitivity** | 0.72(0.56, 0.85) | 0.40(0.28, 0.53) | 0.77(0.61, 0.88) | 0.72(0.56, 0.85) | 1.00(0.99, 1.00) | 0.72(0.56, 0.85) | 0.95(0.84, 0.99) | 0.74(0.59, 0.86) | 0.81(0.67, 0.92) | 0.72(0.56, 0.85) | 1.00(0.99, 1.00) |
| **Specificity** | 0.74(0.62, 0.84) | 0.88(0.78, 0.95) | 0.93(0.84, 0.98) | 0.88(0.78, 0.95) | 1.00(0.98, 1.00) | 0.81(0.70, 0.90) | 0.55(0.43, 0.67) | 0.83(0.72, 0.91) | 1.00(0.95, 1.00) | 0.88(0.78, 0.95) | 1.00(0.98, 1.00) |
| **PPV** | 0.63(0.48, 0.77) | 0.76(0.59, 0.89) | 0.87(0.72, 0.96) | 0.79(0.64, 0.91) | 1.00(0.99, 1.00) | 0.70(0.55, 0.83) | 0.57(0.45, 0.69) | 0.73(0.57, 0.85) | 1.00(0.90, 1.00) | 0.79(0.64, 0.91) | 1.00(0.99, 1.00) |
| **NPV** | 0.81(0.69, 0.90) | 0.61(0.51, 0.71) | 0.86(0.77, 0.93) | 0.84(0.73, 0.91) | 1.00(0.98, 1.00) | 0.82(0.71, 0.91) | 0.95(0.83, 0.99) | 0.84(0.73, 0.92) | 0.90(0.81, 0.95) | 0.84(0.73, 0.91) | 1.00(0.98, 1.00) |
| **PLR** | 2.76(1.78, 4.28) | 3.45(1.69, 7.06) | 10.59(4.48, 25.03) | **6.22(3.16, 12.24)** | Inf(NaN, Inf) | 3.83(2.27, 6.46) | 2.12(1.62, 2.78) | 4.28(2.49, 7.37) | Inf(NaN, Inf) | 6.22(3.16, 12.24) | Inf(NaN, Inf) |
| **NLR** | 0.38(0.23, 0.62) | 0.68(0.55, 0.84) | 0.25(0.15, 0.43) | 0.32(0.19, 0.51) | 0.00(0.00, NaN) | 0.34(0.21, 0.56) | 0.08(0.02, 0.33) | 0.31(0.18, 0.52) | 0.19(0.10, 0.35) | 0.32(0.19, 0.51) | 0.00(0.00, NaN) |

Abbreviations: SVM, supported vector machine; NN, neural network; MLP, Multi-Layer Perceptron; GP, Gaussian Process; GBM, Gradient Boosting Machine; LR, Logistic Regression; NB, Naive Bayes; XGB, XGBoost; C5.0, C5.0 Decision Trees; KNN, k-nearest neighbor; RF, Random Forest; PPV, Positive predictive value; NPV, Negative predictive value; PLR, Positive likelihood ratio; NLR, Negative likelihood ratio;

^a^ All constructed predictive models were developed without the utilization of data augmentation techniques.
